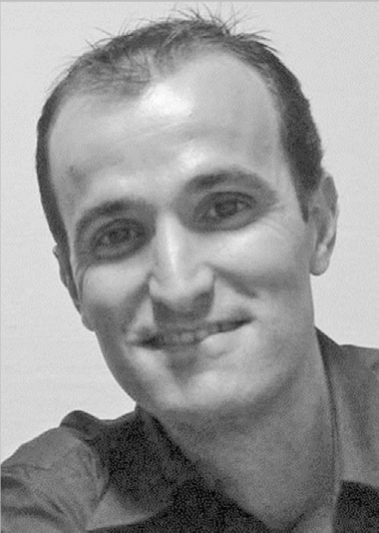# Modern Synthetic Avenues for the Preparation of Functional Fluorophores

**DOI:** 10.1002/anie.201609394

**Published:** 2017-02-17

**Authors:** Fabio de Moliner, Nicola Kielland, Rodolfo Lavilla, Marc Vendrell

**Affiliations:** ^1^ MRC/UoE Centre for Inflammation ResearchThe University of Edinburgh47 Little France CrescentEdinburghEH16 4TJUK; ^2^ Laboratory of Organic Chemistry, Faculty of PharmacyUniversity of BarcelonaBarcelona Science Park, Baldiri Reixac 10-12Barcelona08028Spain; ^3^ CIBER-BBN, Networking Centre for Bioengineering, Biomaterials and NanomedicineBaldiri Reixac 10-12Barcelona08028Spain

**Keywords:** C−H activation, fluorescent probes, imaging, microscopy, multicomponent reactions

## Abstract

Biomedical research relies on the fast and accurate profiling of specific biomolecules and cells in a non‐invasive manner. Functional fluorophores are powerful tools for such studies. As these sophisticated structures are often difficult to access through conventional synthetic strategies, new chemical processes have been developed in the past few years. In this Minireview, we describe the most recent advances in the design, preparation, and fine‐tuning of fluorophores by means of multicomponent reactions, C−H activation processes, cycloadditions, and biomolecule‐based chemical transformations.

## 1 Introduction

**Figure 1:**
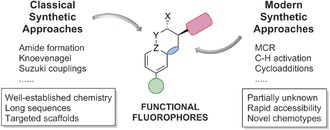
Classical and modern synthetic strategies for the preparation of functional fluorophores.

The search for functional molecules is a pivotal process in many areas of chemistry where structures with well‐defined reactivity and selectivity profiles are needed.^1^ In this context, functional fluorophores are useful tools to interrogate biological processes by targeting very diverse analytes (from small ions to large macromolecules) in complex environments and under physiological conditions. Although standard synthetic approaches with classical reactions have been successful in many cases, the preparation of sophisticated fluorophores cannot always be achieved through well‐established reactions.^2^ Herein, we review the modern strategies that have been developed during the past five years to synthesize functional fluorophores by multicomponent reactions (MCRs), metal‐catalyzed C−H activation, cycloadditions, and biomolecule‐based methods. These strategies have not only accelerated the preparation of unique fluorescent compounds but also enabled the exploration of chemotypes that are inaccessible through conventional approaches (Figure 1).

## 2 Multicomponent Reactions

MCRs constitute a favorable approach for the synthesis of complex molecules given their convergent character, modular features, and access to novel chemotypes.^3^ Müller and Levi recently reviewed the preparation of chromogenic structures by MCRs,^4^ and distinguished between two main strategies, namely 1) the “scaffold approach”, where one of the reactants contains a chromophore, and 2) the “chromophore approach”, where the MCR generates a chromophore from non‐fluorescent materials.

**Figure 2:**
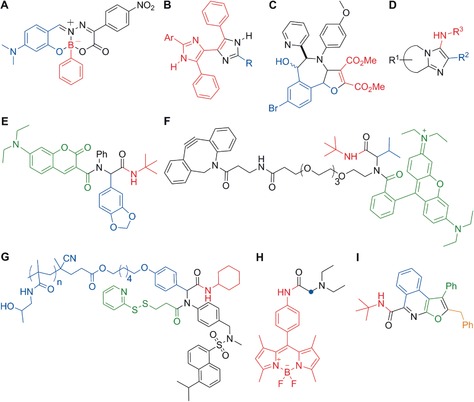
Representative examples of functional fluorophores obtained by MCRs. A) A polarity‐dependent boron‐containing fluorophore. B) Bis(imidazole) heavy‐metal probes. C) N‐Heterocyclic carbene derived optical probes. D) Fluorophores that bind to benzodiazepine receptors in mitochondria. E) Coumarin‐containing fluorescent peptoids that target mitochondria. F) Rhodamine‐based tags for bioorthogonal chemistry and protein profiling. G) Dansyl‐based protein reactive polymers. H) PhagoGreen as a pH‐sensitive BODIPY fluorophore for in vivo imaging of phagocytic macrophages. I) Blue‐emitting furoisoquinolines. The fragments originating from the various precursor building blocks are colored to highlight the multicomponent nature of the syntheses.

Several examples of the chromophore approach have been reported. The group of Pischel and Gois developed a three‐component sequential condensation reaction of a boronic acid, a salicylaldehyde, and an amino substrate to obtain fluorescent boron‐containing heterocycles with polarity‐dependent fluorescence emission (Figure 2 A).^5^ These structures were suitable for the imaging of dendritic cells as well as cancer cells. As a representative example of fluorophores obtained from more conventional condensation reactions, Palumbo Piccionello and co‐workers described a three‐component reaction (3‐CR) of an imidazole‐substituted dicarbonyl compound with aldehydes and an ammonia source to form 4,4′‐bis(imidazole)s as selective fluorescent probes for heavy metals (Figure 2 B).^6^ Cheng and co‐workers exploited N‐heterocyclic carbene chemistry to prepare fluorophores in MCRs (Figure 2 C).^7^ The reaction of imidazopyridinium salts as carbene precursors, phthalaldehydes, and dimethyl acetylenedicarboxylate (DMAD) afforded benzofuroazepines with emission wavelengths in the visible range (ca. 500 nm) and high fluorescence quantum yields (up to 81 %).

Metal‐catalyzed MCRs represent a large portion of the reactions applied in the chromophore approach.^8^ In this respect, a broad range of fluorophores can be accessed through sequential Müller‐type processes, which involve a palladium‐catalyzed Sonogashira coupling in combination with cascade cyclizations, cycloadditions, and/or additional condensation reactions. Libraries of structurally diverse merocyanines, imidazoles, indolones, furans, oxazoles, thiophenes, quinolones, and other heterocyclic systems have been prepared, providing an excellent chemical platform to fine‐tune the photophysical and biological properties of fluorescent compounds.

Isocyanide‐based MCRs are the most exploited family of multicomponent condensations. These reactions rely on the versatility of isocyanide species, which can engage with multiple counterparts. An interesting example towards high‐throughput chemistry was reported by Balakirev and co‐workers, who made use of the Groebke–Blackburn–Bienaymé reaction between heterocyclic amidines, aldehydes, and isocyanides to generate a library of 1600 compounds in droplet arrays (Figure 2 D).^9^ Subsequent analysis identified fluorophores with emission wavelengths ranging from 485 nm to 627 nm, and selected fluorophores were used to image the mitochondrial benzodiazepine receptor TSPO in PC3 prostate cancer cells, among others.

The Ugi 4‐CR, which combines isocyanides with aldehydes, amines, and carboxylic acids, is a particularly suitable method for the synthesis of fluorescent structures through the scaffold approach. Neto, da Silva, and co‐workers used a coumarin carboxylic acid to prepare a collection of fluorescent adducts with high affinity for mitochondria (Figure 2 E).^10^ Likewise, Westermann and co‐workers described a sophisticated family of fluorescent tags for protein profiling from an Ugi 4‐CR with rhodamine B as the carboxylic acid fluorophore (Figure 2 F).^11^ The Ugi approach has also been successfully applied to polymeric structures. Multifunctional fluorescent polymers for avidin and bovine serum albumin conjugation were prepared through an Ugi MCR in combination with a controlled reversible addition–fragmentation chain‐transfer polymerization (Figure 2 G).^12^ An example of the applicability of Ugi 4‐CRs to functionalize fluorophores for optical imaging was reported by our group.^13^ We described the preparation of a fluorescent isocyanide–BODIPY core and its derivatization using different MCRs. Subsequent biological analysis identified PhagoGreen as a pH‐sensitive fluorophore for imaging phagocytic macrophages in vivo (Figure 2 H). An extension of this approach has been reported by Peña‐Cabrera and co‐workers with the derivatization of aldehyde‐functionalized BODIPYs in a Passerini MCR.^14^ With regard to the chromophore approach, Riva, Müller, and co‐workers recently described an Ugi MCR in which the initial adducts were converted into furo[2,3‐*c*]isoquinolines in a palladium‐catalyzed insertion/alkynylation/cycloisomerization cascade.^15^ The resulting isoquinolines displayed strong fluorescence, with emission maxima ranging from 396 nm to 443 nm and tunable quantum yields depending on the substituents (Figure 2 I).

**Figure 3:**
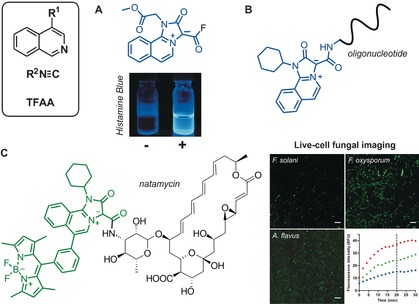
Synthesis and application of acyl fluoride mesoionic fluorophores. Isoquinoline‐based mesoionic fluorides for the A) fluorescence detection of histamine (Histamine Blue) and B) fluorescence labeling of oligonucleotides. C) Green‐fluorescent mesoionic BODIPY conjugated to natamycin for imaging fungal cells. Fluorescence images of different fungal species after incubation with the natamycin analogue (5 μm) for 20 min and time‐course analysis. Scale bars: 20 μm. *F. oxysporum* red, *A. flavus* green, *F. solani* blue. TFAA=trifluoroacetic anhydride. Reproduced with permission from the Royal Society of Chemistry^17^ (A) and the American Chemical Society (C).^19^.

Occasionally, unusual reactivity patterns in reactions with isocyanides can lead to unprecedented structures. An interesting example is the preparation of blue‐fluorescent mesoionic acid fluorides from isocyanides, azines, and fluorinated anhydrides.^16^ These mesoionic acid fluorides were found to be remarkably stable to hydrolysis, and were employed for imaging histamine in live cells^17^ as well as for labeling oligonucleotides^18^ (Figure 3 A and B, respectively). Recently, this approach has been extended to isoquinoline‐substituted BODIPY structures. The resulting mesoionic BODIPY compounds were used for the activation‐free labeling of bioactive amines, and a fluorescent analogue of the antimycotic agent natamycin was developed for imaging fungal cells (Figure 3 C).^19^

Finally, Li and co‐workers described a dipolar isocyanide‐based MCR to produce complex pyrrolophenanthrolines under solvent‐free conditions in excellent yields from isocyanides, aldehydes, malononitriles, and phenanthrolines.^20^ The adducts showed a selective increase in fluorescence emission upon incubation with Cu^2+^, showing potential for the detection of metal ions in biological assays.

## 3 Metal‐Catalyzed C−H Activation Reactions

Metal‐catalyzed couplings, such as Suzuki–Miyaura reactions, are the most common approach to prepare biaryl compounds. However, the need for two functionalized substrates, such as a boronic acid and an aryl halide, often represents a limitation owing to the restricted availability of substituted boronic acid derivatives. These limitations can be overcome with C−H activation processes that directly connect aryl halides to (hetero)arenes by metal‐promoted activation of a C−H bond in the latter compound.^21^

**Figure 4:**
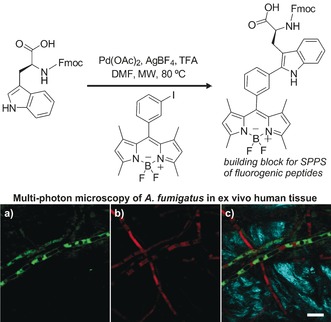
Top: Synthesis of a fluorogenic Trp–BODIPY amino acid by C−H activation. The Trp–BODIPY amino acid was incorporated into antimicrobial peptides to image the fungal pathogen *A. fumigatus* in ex vivo human tissue by multi‐photon microscopy. Bottom: Fluorescence images of a) the fluorogenic peptide, b) RFP‐expressing *A. fumigatus*, and c) merged (a) and (b) together with the second harmonic generation from collagen fibers from lung tissue. Scale bar: 10 μm. DMF=*N*,*N*‐dimethylformamide, Fmoc=9‐fluorenylmethoxycarbonyl, MW=microwave, SPPS=solid‐phase peptide synthesis, TFA=trifluoroacetic acid. Reproduced with permission from Springer Nature.^22^

In this context, we have recently described the straightforward synthesis of a fluorogenic tryptophan (Trp) based amino acid as a key building block for the preparation of peptide‐based fluorophores.^22^ The amino acid was prepared in a single step and in good yields by coupling *meta*‐iodophenyl‐substituted BODIPY and Fmoc‐Trp‐OH in the presence of Pd(OAc)_2_ under microwave irradiation (Figure 4, top). Afterwards, the Trp–BODIPY amino acid was incorporated into antimicrobial peptides to label the fungal pathogen *Aspergillus fumigatus* in ex vivo human tissue (Figure 4, bottom). Notably, the peptide labeling did not compromise their activity and selectivity, creating numerous opportunities for the development of novel peptide‐based imaging probes.

Ackermann and co‐workers have developed a method for the arylation of short peptides that is based on the use of hypervalent iodoaromatic species in palladium‐catalyzed C−H activation processes.^23^ Hansen and co‐workers described the gold‐catalyzed chemoselective ethynylation of Trp‐containing peptides and proteins^24^ for subsequent fluorophore conjugation by click chemistry.

**Figure 5:**
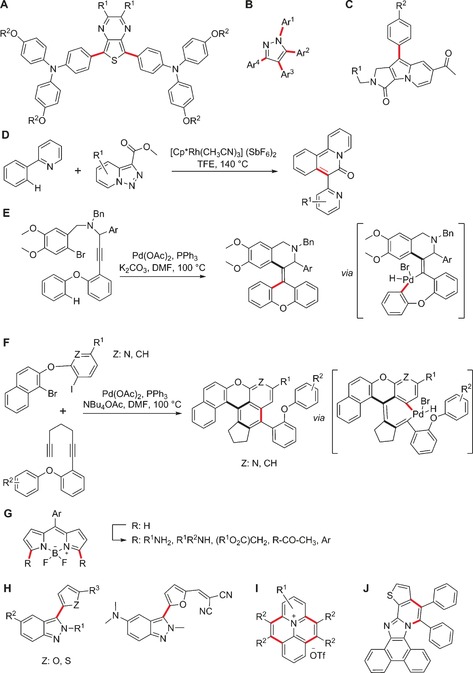
Preparation of fluorescent compounds by C−H activation. A) Donor–acceptor–donor thienopyrazines as NIR dyes. B) Tunable tetraaryl pyrazole fluorophores. C) Seoul‐Fluors (pyrroloindolizinone fluorophores). D) Pyridotriazole approach to metal probes. E) Palladium‐catalyzed cascade reactions towards solid‐state‐emitting xanthenes. F) A C−H activation approach to fluorescent polyheteroaromatic systems. G) C−H activation to derivatize the BODIPY core. H) CDCs towards indazole‐based fluorescent dyads. I) Fluorescent probes obtained by functionalization of *N*‐phenylpyridinium ions. J) An Fe^3+^ probe obtained by two CDCs. The chemical bonds formed upon C−H activation are highlighted in red. OTf=trifluoromethanesulfonate, TFE=2,2,2‐trifluoroethanol.

C−H activation has also been successfully employed for the functionalization of other heterocycles, as exemplified in the recent work of Delcamp, Hammer, and co‐workers.^25^ Fluorescent thienopyrazine‐based donor–acceptor–donor compounds were prepared by double C−H arylation of the thiophene moiety. The resulting adducts displayed large Stokes shifts with emission in the near‐infrared (NIR) region (Figure 5 A). Another modular C−H activation strategy was described for the preparation of highly substituted pyrazoles. Four sequential palladium‐catalyzed direct arylations enable the synthesis of fluorescent tetraaryl pyrazoles with emission maxima between 389 nm and 439 nm (Figure 5 B).^26^ C−H activation approaches have also been used to fine‐tune the photophysical properties of functional fluorophores. Park and co‐workers reported the preparation of a collection of pyrroloindolizinones (Seoul‐Fluors, Figure 5 C); the fluorescence quantum yields were systematically explored by derivatizing the central scaffold with different aryl groups in palladium‐catalyzed couplings.^27^ Furthermore, these results were exploited to develop new fluorophores for imaging reactive oxygen species in human cancer cells.^27^

Aside from accelerating the diversification and optimization of fluorophores, C−H activation can be also employed to generate complex fluorescent structures from very simple precursors. Glorius and co‐workers achieved the synthesis of polycyclic frameworks in a single step through a rhodium(III)‐catalyzed coupling between an aryl pyridine and a pyridotriazole as a carbene precursor (Figure 5 D).^28^ Notably, the pyridyl moiety plays a dual role; during the synthesis, it stabilizes the intermediates and coordinates the catalyst while it later functions as a fluorescent reporter by enabling the detection of metal ions. Spectroscopic analysis of these structures revealed a blue shift in the absorption and emission maxima in the presence of Cu^2+^ or Zn^2+^ ions, making them potentially useful for the detection of metal ions.

Another interesting strategy to construct fluorophores by means of C−H activation is to incorporate such activation processes into domino pathways, where they take place alongside other bond‐forming reactions. Perumal and Nandakumar recently reported a two‐step, one‐pot palladium‐catalyzed carbopalladation/C−H activation method for the generation of xanthene derivatives featuring a tetrasubstituted olefin in high yields (Figure 5 E).^29^ These fluorophores are non‐emissive in organic solvents, but exhibit pronounced green to yellow fluorescence with large Stokes shifts in water and thus constitute an example of aggregation‐induced emission. Tietze and co‐workers prepared a sophisticated fluorescent polyheteroaromatic scaffold through a palladium‐catalyzed cascade process encompassing one Sonogashira coupling followed by double carbometalation of triple bonds and a final C−H arylation (Figure 5 F).^30^ On the other hand, Dehaen and co‐workers have extensively derivatized the fluorescent BODIPY core by C−H activation reactions—making use of either radical chemistry or palladium catalysis—to produce collections of BODIPY fluorophores (Figure 5 G).^31^

The applicability of C−H activation can be extended by including cross‐dehydrogenative couplings (CDCs), oxidative transformations linking two substrates through double C−H activation processes that do not require any functionalized precursors.^32^ These approaches are limited by the ubiquity of C−H bonds but the recent discovery of new selectivity rules is enabling the rapid expansion of the field. For instance, CDCs have been used by You and co‐workers to tune donor–acceptor dyads by linking electron‐rich (e.g., furans, thiophenes) and electron‐poor heterocycles (e.g., indazoles) in regioselective oxidative couplings (Figure 5 H).^33^ The resulting bis(heteroaryl) dyes (Indazofluors) display full‐color‐tunable emission (393–725 nm), high fluorescence quantum yields (up to 93 % in CH_2_Cl_2_, relative to rhodamine B),^33^ and could find applications as subcellular organelle markers.

Alkyne annulations are another type of CDCs in which carbon–carbon triple bonds and two atoms of a suitable partner react to form carbo‐ or heterocycles. For instance, Cheng and co‐workers described rhodium‐catalyzed annulations of 2‐aryl pyridines and alkynes under O_2_ atmosphere to prepare fluorescent pyridinium salts with potential applications in organic electronic devices.^34^ Similarly, Wang and co‐workers prepared polycyclic quinolinium cations in double C−H activation/annulation processes (Figure 5 I).^35^ Hua and Zheng reported oxidative coupling reactions with [(RhCl_2_Cp*)_2_] (Cp*=pentamethylcyclopentadienyl) as the catalyst to obtain complex heterofused phenanthroimidazoles in very good yields (Figure 5 J).^36^ In this example, the synthetic protocol was extended to C−H/N−H activation of the heterocyclic input, and led to new fluorescent ratiometric probes for Fe^3+^ ions. Finally, palladium‐mediated C−H/N−H activation methods have been used by Kundu and co‐workers for the synthesis of pyrido[1,2‐*a*]indoles with high fluorescence quantum yields, tunable emission (478–588 nm), and properties suitable for cell imaging.^37^

## 4 Cycloaddition Reactions in Fluorescent Probe Development

**Figure 6:**
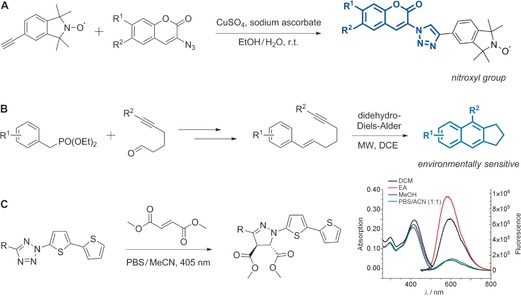
Synthesis of functional fluorophores by means of cycloadditions. A) Preparation of redox‐sensitive coumarins by azide–alkyne 1,3‐dipolar cycloaddition. B) Solvatochromic naphthalenes obtained by intramolecular didehydro‐Diels–Alder reactions. DCE=1,2‐dichloroethane. C) Photoclick reaction between tetrazoles and alkenes to generate environmentally sensitive fluorophores. Inset: Absorbance and fluorescence (*λ*_ex_=405 nm) spectra of a representative pyrazoline adduct in different solvents. ACN=acetonitrile, DCM=dichloromethane, EA=ethyl acetate, PBS=phosphate‐buffered saline. Reproduced with permission from the American Chemical Society.^48^

Cycloaddition reactions are a valuable strategy to access highly functionalized structures owing to their experimental ease, good synthetic yields, and compatibility with multiple functional groups,^38^ with the alkyne–azide 1,3‐dipolar Huisgen cycloaddition^39^ being one of the most widely used reactions in chemical biology. This strategy was utilized by Fairfull‐Smith and co‐workers to conjugate azidocoumarin derivatives to an alkyne‐containing isoindoline nitroxyl in a copper‐catalyzed azide–alkyne cycloaddition (CuAAC) process to generate fluorophores with high sensitivity to oxidative processes (Figure 6 A).^40^ Another example is the preparation of highly decorated squaraine rotaxane dendrimers by Smith and co‐workers.^41^ In this case, a squaraine fluorescent core was encapsulated within a macrocycle containing four alkyne groups that were clicked to azido amines to achieve bright deep‐red fluorophores with high photostability. Together with the Chang group, our group has also adapted CuAAC reactions for the synthesis of diversity‐oriented fluorescence libraries^42^ to optimize the spectral properties of functional fluorophores^43^ and their binding capabilities to specific biological targets.^44^

Cycloaddition reactions employing heterocyclic or carbocyclic partners have also been described within the realm of fluorophore synthesis. The use of didehydro‐Diels–Alder reactions for the preparation of environmentally sensitive probes (Figure 6 B) was recently summarized by Brummond and Kocsis.^45^ Such processes can afford dihydronaphthalene and naphthalene fluorophores resembling the solvatochromic dye Prodan. Ishii and co‐workers reported the synthesis of 1,4‐diaryl‐1‐thio‐1,3‐butadienes with π‐donor and π‐acceptor groups by intramolecular [4+2] cycloadditions of 1‐thio‐enynes linked to an anthracene ring.^46^ These polycyclic frameworks fluoresced in the red and NIR regions and exhibited marked solvatochromism.

The tetrazole–alkene photoclick reaction is an unusual [3+2] cycloaddition, taking place upon photoirradiation and producing pyrazolines via a transient nitrile imine. The preparation of profluorescent nitroxides by UV irradiation of isoindoline oxide tetrazoles and maleimides was recently reported by Barner‐Kowollik, Blinco, and co‐workers.^47^ In these molecules, a stable free radical moiety was covalently tethered to a fluorophore so that the nitroxide radical quenched the fluorescence, and emission was only detected in the presence of radicals. Another example was published by Lin and co‐workers with the reaction between tetrazoles containing extended π‐systems and dimethyl fumarate upon irradiation at 405 nm (Figure 6 C).^48^ The resulting pyrazoline cycloadducts displayed significant bathochromic shifts in organic solvents when compared to aqueous media, suggesting their potential application as environmentally sensitive fluorophores.

The involvement of several reaction centers has been exploited to expand the versatility and complexity of fluorescent structures. For instance, rhodium‐catalyzed [2+2+2] cycloadditions of biaryl‐linked diynes with alkynes, nitriles, and isocyanates can afford triphenylenes and azatriphenylenes with broad emission ranges (359–498 nm) and high quantum yields (up to 88 %) in a single operation.^49^ Cycloadditions can be also combined with other reactions, such as oxidations, in one‐pot transformations. An intramolecular [2+2+2] strategy entailing the cyclization of bis(propargylphenyl)carbodiimides under rhodium catalysis followed by oxidative aromatization was reported by Saito and co‐workers.^50^ L‐shaped penta‐, hexa‐, and heptacycles with a pyrrolo[1,2‐*a*][1,8]naphthyridine unit were prepared in a one‐pot process as fluorophores with blue to orange emission. Alkynes have also been reacted with pyridoisoindoles and pyrrolopyridines to generate indolizines by intermolecular thermal cycloaddition and DDQ‐promoted oxidation (DDQ=2,3‐dichloro‐5,6‐dicyano‐1,4‐benzoquinone).^51^

Domino reactions are intrinsically useful as they allow to explore broad structural diversity in a highly efficient manner. These processes can be used for the simultaneous formation of several bonds and, together with cycloaddition reactions, have enabled the assembly of synthetically challenging fluorophores. Diederich and co‐workers reported the synthesis of complex tetracenes from cumulenes through a [2+2] cycloaddition of tetracyanoethylene to the central double bond of the cumulene structure, followed by electrocyclization, dehydrogenation, and final copper‐promoted thermal oxidation.^52^ Notably, the resulting structures showed fluorescence changes after binding to metal ions, such as Cu^+^ and Ag^+^. Another cascade‐based approach was recently developed by Wender and co‐workers, with a domino sequence rendering polycyclic compounds through [4+2] cycloaddition and elimination followed by a second [4+2] cycloaddition.^53^ This approach yielded solvatochromic tetracyclic dyes after two additional functionalization steps.

**Figure 7:**
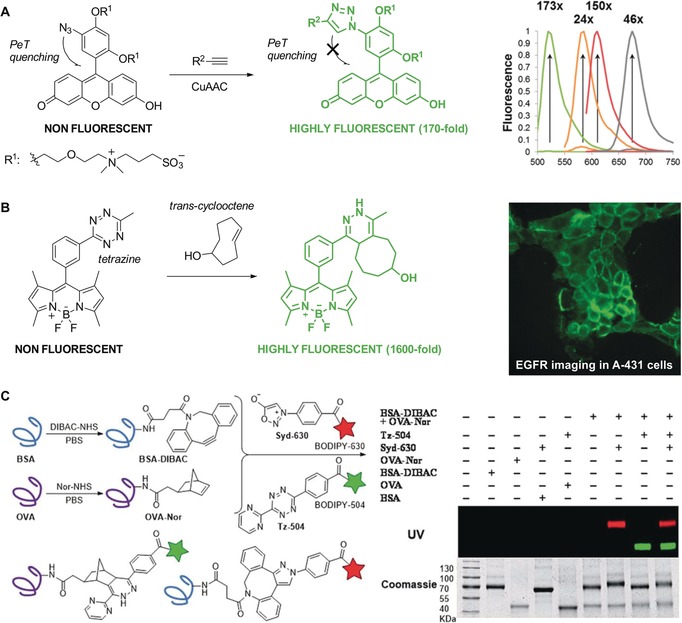
Fluorogenic probes with emission enhancement upon cycloaddition. A) Fluorescein‐based Calfluors with intramolecular photoinduced electron transfer (PeT) quenching and fluorescence emission after CuAAC reaction. Fluorescence spectra of Calfluors covering the whole spectral range. B) A representative example of a BODIPY–tetrazine fluorogen and its Diels–Alder condensation with *trans*‐cyclooctenes (TCO) to produce superbright fluorophores. Fluorescence microscopy images of A‐431 live cells after incubation with anti‐EGFR TCO‐conjugated monoclonal antibodies and BODIPY–tetrazines. C) Modifications of protein surfaces by cycloadditions with sydnones. Left: Dibenzoazacyclooctyne (DIBAC) and 5‐norbornene‐2‐acetic acid (Nor) are attached to the proteins BSA and OVA by amide formation. The labeled proteins BSA–DIBAC and OVA–Nor simultaneously react with sydnone–BODIPY (Syd‐630) and tetrazine–BODIPY (Tz‐504). Right: Gel analysis of BSA–DIBAC and OVA–Nor after incubation with either Syd‐630, Tz‐504, both reagents simultaneously, or no reagent (−). Reproduced with permission from the American Chemical Society^54^ (A), Wiley‐VCH^56^ (B), and the Royal Society of Chemistry^57^ (C).

Cycloaddition reactions have been also adapted to bioorthogonal chemistry to prepare fluorogenic structures that undergo a fluorescence enhancement upon reaction with their counterparts. Bertozzi and co‐workers described the synthesis of “Calfluor” fluorogens with emission maxima covering the entire visible spectrum (Figure 7 A).^54^ Conveniently, Calfluors are internally quenched by azide groups so that their fluorescence emission is turned on after a click reaction with suitable alkynes. This feature enables their application for imaging under no‐wash conditions in cells, tissues, and zebrafish. Strained cyclic alkynes have been developed to avoid the need for copper in cycloaddition reactions in biological systems. Boons and co‐workers investigated cycloadditions of the dibenzocyclooctyne derivative FI‐DIBO with different partners (e.g., azides, nitrones, nitrile oxides, diazo derivatives) under catalyst‐free conditions, and obtained 1*H*‐pyrazole fluorophores with 160‐fold fluorescence enhancement over FI‐DIBO.^55^ Alternatively, tetrazines can be coupled to strained cyclic olefins, such as norbornenes or *trans*‐cyclooctenes. Weissleder and co‐workers reported the synthesis of non‐fluorescent tetrazine–BODIPY dyes showing 1600‐fold fluorescence enhancement after their reaction with *trans*‐cyclooctenol (Figure 7 B).^56^ The authors validated the biological application of these fluorophores by visualizing intracellular and extracellular targets in both fixed and live cells. Finally, the groups of Houk and Murphy described the coupling of the mesoionic heterocycle sydnone to fluorophores amenable to cycloadditions with strained cyclooctynes.^57^ In addition to displaying good reactivity under physiological and catalyst‐free conditions, these couplings proved to be orthogonal to the reactions between tetrazines and norbornenes (Figure 7 C).

## 5 Biomolecule‐Based Chemical Transformations

The need for sophisticated probes in chemical biology has prompted the adaptation of new chemical strategies to interrogate biological systems under physiological conditions. In this Section, we review some recent examples of biology‐oriented modern chemical transformations of functional fluorophores and their application at the interphase between chemistry and biology.

Functional fluorophores have been broadly applied to investigate different biomolecules, from small‐molecule drugs to large enveloped viruses.^58^ Weissleder and co‐workers described a *trans*‐cyclooctene‐modified taxol analogue and its localization in intracellular tubules upon reaction with activatable tetrazine‐linked fluorophores.^59^ Notably, the high rate of these reactions (ca. 1000 m ^−1^ s^−1^ at 37 °C) renders them an optimal approach for identifying binding targets of tagged drugs in live cells. More recently, DeRose and co‐workers modified the chemotherapeutic drug picoplatin with an azide group to identify and image its oligonucleotide binding targets upon conjugation with alkyne‐derivatized dansyl fluorophores.^60^ A similar strategy was used by Wnuk and co‐workers to modify nucleosides and nucleotides with azido groups and couple them to strained cyclooctynes for direct imaging in MCF‐7 cancer cells.^61^ The nucleobase–triazole adducts proved to be suitable for fluorescence lifetime imaging of specific signaling events inside live cells.

Metabolic signaling is an important area in the life sciences, which has become much more accessible thanks to the development of bioorthogonal functional fluorophores. After the seminal work with metabolically compatible glycans,^62^ Bertozzi and co‐workers exploited the metabolic incorporation of UDP‐4‐azido‐4‐deoxyxylose (UDP=uridine diphosphate) to study the function of glycosaminoglycans (GAGs) in zebrafish embryogenesis.^63^ The in vivo coupling of these sugars to fluorescent cyclooctynes revealed new links between GAG abundance and embryonic development. Additional work has resulted in the adaptation of cycloaddition reactions for advanced imaging technologies, such as two‐photon and fluorescence lifetime imaging,^64^ and the ratiometric visualization of alkyne‐modified metabolites in live cells.^65^

**Figure 8:**
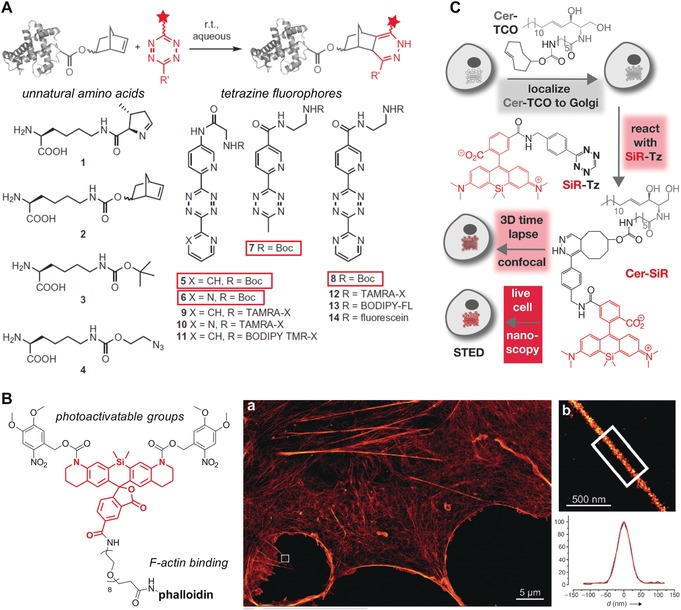
Functional fluorophores using biomolecule‐based approaches. A) Protein labeling by inverse‐electron‐demand Diels–Alder cycloadditions. Structures of genetically encoded unnatural amino acids and tetrazine‐containing fluorophores. B) Photoactivatable phalloidin conjugate of 5‐carboxy‐NVOC2‐SiRhQ. a) Super‐resolution microscopy image of a COS‐7 cell stained with the phalloidin conjugate. b) Expanded image of the boxed region in (a), showing a protruding filopodial structure, and the line‐scan intensity across the filopodial structure in (b) (shown in black) and a Gaussian fit (red). C) Two‐step procedure for subcellular labeling of the Golgi apparatus in live cells; cells are treated first with Cer‐TCO, a *trans*‐cyclooctene‐containing ceramide lipid, and then reacted with the tetrazine fluorophore SiR‐Tz for 3D confocal and stimulated emission depletion (STED) super‐resolution microscopy. Reproduced with permission from Springer Nature^68^ (A) and Wiley‐VCH^74^, ^77^ (B, C).

Similar chemical transformations have been applied to peptide‐based structures, after the pioneering work from the Schultz group on the genetic code expansion technology.^66^ Lemke and co‐workers described a set of strained dienophilic unnatural amino acids that could be incorporated into proteins through suppression of the amber stop codon.^67^ The conjugation of these amino acids to tetrazine fluorophores enabled direct protein labeling in live cells in an orthogonal manner to cyclooctyne–azide chemistry. Chin et al. described the genetic encoding of norbornene amino acids in both *E. coli* and mammalian cells for exceptionally fast and site‐specific protein labeling upon reaction with tetrazines (Figure 8 A).^68^ This work has later been extended to other functional groups, such as the phenylsydnone 1,3‐dipole and bicyclononyne pair, for strain‐promoted reactions under physiological conditions.^69^ Kele and co‐workers recently published strain‐promoted azide–alkyne cycloadditions in peptide sequences.^70^ The authors synthesized a quenched bis(azide) fluorogenic probe for two‐point binding tagging of bis‐cyclooctynylated short hexapeptides in the pursuit of self‐labeling small peptide tag motifs.

Devaraj and co‐workers described cycloaddition transformations on oligonucleotides to improve the detection of specific DNA or RNA sequences in genomic analysis and diagnostics. The authors initially developed fluorescent DNA structures with quenched tetrazine fluorophores and methyl cyclopropenes that “clicked” only in the presence of complementary sequences.^71^ More recently, these nucleic acid templated reactions between 7‐azabenzonorbornadiene and fluorogenic tetrazines have been optimized to detect DNA and microRNA templates in picomolar concentrations.^72^

Another area of biological research that has strongly benefited from new synthetic approaches towards functional fluorophores is super‐resolution microscopy. The Lavis group has been a major contributor in this field and recently described the incorporation of four‐membered azetidine rings into fluorescent scaffolds as a simple structural modification to improve the brightness and photostability of dyes.^73^ Moreover, some recent work on rhodamine structures has led to caged Si‐rhodamine fluorophores as photoactivatable labels for super‐resolution imaging (Figure 8 B).^74^ Such functional fluorophores have been prepared by means of cycloaddition reactions using the above‐mentioned approaches. For instance, Chin and co‐workers recently reported super‐resolution stochastic optical reconstruction microscopy (STORM) imaging of cytoskeletal proteins (e.g., β‐actin, vimentin) after introducing bicyclo[6.1.0]nonyne‐functionalized lysine residues at specific sites and coupling them with tetrazine fluorophores.^75^ The enhanced resolution achieved with these technologies has enabled the visualization of dynamic processes in specific subcellular compartments, such as single‐molecule tracking of *N*‐sialic acids and O‐linked *N*‐acetylgalactosamine in live cells,^76^ and prolonged live‐cell imaging of the Golgi apparatus by STED microscopy (Figure 8 C).^77^

## 6 Summary and Outlook

Selective and non‐invasive imaging of biologically relevant targets represents a major challenge in the life sciences. Probes that are able to meet these requirements tend to have sophisticated molecular frameworks, which are often at the limit of our synthetic capability. Well‐established reactions are robust and practical but might only provide access to a restricted chemical space. These synthetic challenges have prompted the development of modern chemical methods to generate fluorescent structures with optimal properties. Synthetic methods such as C−H activation, multicomponent, or cycloaddition reactions have proven extremely useful to develop new functional fluorophores as well as to optimize their spectral features and integrate them into advanced imaging technologies, such as super‐resolution microscopy. These approaches constitute an excellent synthetic platform and complement currently available methods to design the next generation of fluorophores for biomedical research.

## Biographical Information

*Fabio de Moliner obtained his Ph.D. in Organic Chemistry from the University of Genova in 2010 under the guidance of Prof. Andrea Basso. After graduation, he joined the research group of Prof. Christopher Hulme at the University of Arizona (Tucson) as a postdoctoral research assistant to focus on the design and application of chemical methods based on multicomponent and domino processes. He joined the Vendrell group at the University of Edinburgh in 2015. His main research interests are the use of fluorescent scaffolds to investigate the transport of xenobiotics and the development of optical probes for in vivo imaging*.

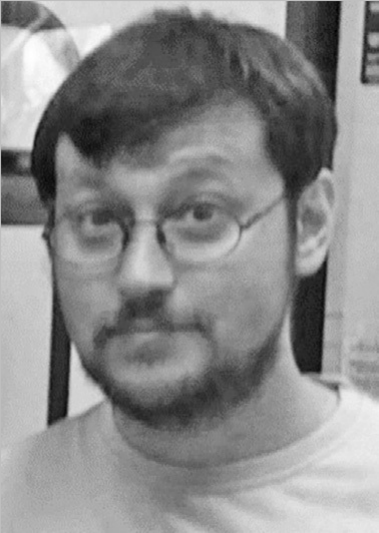

## Biographical Information

*Nicola Kielland graduated with an M.Sc. in Chemistry from the University of Genova in 2006, working on multicomponent reactions (MCRs) under the supervision of Prof. Luca Banfi. He obtained his Ph.D. at the University of Barcelona in 2011 in the Lavilla group, where he developed new MCRs and studied their applications in biomedicine. After a one‐year postdoctoral experience on catalyzed reactions employing carbon dioxide with Prof. Arjan Kleij (ICIQ, Tarragona), he moved back to the University of Barcelona where he is currently working on the development of new probes from multicomponent adducts in the Lavilla group*.

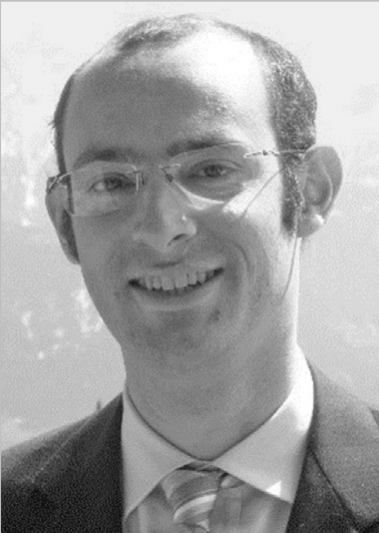

## Biographical Information

*Rodolfo Lavilla studied Pharmacy at the University of Barcelona, where he subsequently obtained his Ph.D. in Organic Synthesis under the supervision of Prof. Mercedes Alvarez. After a postdoctoral stay at the University of California San Diego (Prof. Ernest Wenkert), he returned to the University of Barcelona where he is now Full Professor of Organic and Medicinal Chemistry. His research interests deal with heterocyclic chemistry, multicomponent reactions, and selective peptide modifications*.

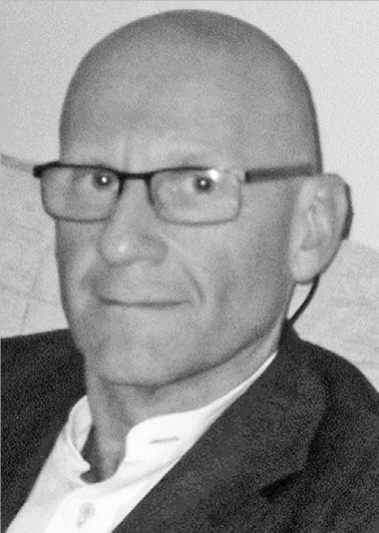

## Biographical Information

*Marc Vendrell obtained his Ph.D. in Chemistry at the University of Barcelona in 2007. He then joined the Singapore Bioimaging Consortium to work with Prof. Young‐Tae Chang on synthetic fluorophores for optical imaging. In 2012 he started his independent career as an MRC Academic Fellow at the University of Edinburgh. He is now a Lecturer in Biomedical Imaging, and his main research interest is the development of activatable fluorophores for imaging cancer and inflammation. His research has been recognized with several international awards, such as the SEQT Young Investigator Award (2007), the SBIC Chairman's Prize (2010), and a Marie Curie CIG (2013)*.